# Supplementary material for: Lower promoter activity of the ST8SIA2 gene has been favored in evolving human collective brains
Source: PLoS One. 2021 Dec 16;16(12):e0259897. doi: 10.1371/journal.pone.0259897 (PMC8675693; doi:10.1371/journal.pone.0259897)
Supplement: S1 Fig — The three promoter SNPs are highlighted in yellow. The “N”s represent uncertain sites. The name of the AMH haplotype is shown by the ID of the individual who has the sequence (phase 3 of the 1000 Genomes Project database [16]). The six SNPs used for the estimation of variant age are highlighted in orange. (PDF) [file pone.0259897.s001.pdf]

```
Chimp            CGGCCCTA--TCAGTTCATGAGCTGGA-A-TGAGGCTATGGTCCTGACGGTTTGCGATTCAAGAGTCTTC-CCCCTATCGTCGCGCGGGCAGCCA
Vindija-1        N...T.NNNN.N.N.N.......GA..N.C......C.......C..............N...G......T...TC.CA...C.CT...G..NNN
Vindija-2        N...T.NNNN.N.N.N........A.GN.C.A....C...A..TC......C.......N...G......T...TC.CA...C..TA..G..NNN
Altai            N...T.NNNN.N.N.N........A.GN.C.A....C...A..TC......C.......N...G......T...TC.CA...C..TA..G..NNN
Chagyrskaya-1    N...T.NNNN.N.NCN.......GA.GN.-......C.......C......C.......N...G......-...TC.CA...C..T...G..NNN
Chagyrskaya-2    N...T.NNNN.N.N.N........A.GN.-.A....C...A..TC......C.......N...G......-...TC.CA...C..TA..G..NNN
Denisovan-1      N...T.NNNN.N.N.N........A.GN.C......C...AG.TCA...........C.N...GA...C.A....C.C....C.........NNN
Denisovan-2      N...T.NNNN.N.N.N........A.GN.C.A....C...A..TC......C.......N...G......A...TC.CA...C..TA..G..NNN
HG04042.1        ....T..GGA....C.T......GA.GA.C......C.......C......C...........G......T...TC.CA...C..T...G.....
HG03772.1        ....T..GGA....C........GA.GA.C......C.......C......C...........G......T...TC.CA...C..T...G..G..
HG03667.1        ....T..GGA....C........GA.GA.C......C.......C......C...........G......T...TC.CA...C..T...G.....
HG02494.1        ....T..GGG....C.T......GA.GA.C......C.......C......C...........G......T...TC.CA...C..T...G.....
NA19664.0        ....T..GGG....C........GA.GA.C......C.......C......C...........G......T...TC.CA...C..T...G.....
HG01455.0        ....T..GGG....C.T......GA.GA.C......C.......C......C.........T.G......T...TC.CA...C..T...G.....
HG01073.1        .AA..TC.AGCTG..G........A..ACC..CC.TCTGA........A......AC.C.G...AC.G.TT..TT.GC...T.A...CA.G....
NA19908.0        ....T..GGA.........CGAT.A..A.C..C..T.TG........TA.C...TAC.C.G.C..C...TT...T.GC...T.A...CA.GA...
NA19472.1        T...T..GGG..G....CC.....CA.G.C......CT......C....A..........G....C...TTT..T.GC...T.A...CA.G....
NA19468.0        .AA..TCGAGCTG..G........A..ACC..CC.TCTGA........A......AC.C.G...AC.G.TT..TT.GC...T.A...CA.G....
NA19116.1        ....T..GGA...T.....CGAT.A..A.C..C..TCTG........TA.C...TAC.C.G.C..C...TT...T.GC...T.A...CA.GA...
NA18909.0        T...T..GGG..G....CC.....CA.G.C......CT......C....A..........G....C...TTT..T.GC...T.A...CA.GA...
HG03572.1        ....T..GGA.........CGAT.A..A.C..C..T.TG........TA.C...TAC.C.G.C..C...TT...T.GC...T.A...CA.G....
HG03437.1        ...AT..GGA..............A.GA.C....T.CT....T...G....CC.......G.........T.T.T.GC...T.A...CA.G....
HG03311.0        ....T..GGA.........CGAT.A..A.C..C..TCTG........TA.C...TAC.C.G.C..C...TT...T.GC...T.A...CA.GA...
HG03105.0        ....T..GGG.........CGAT.A..A.C..C..T.TG........TA.C...TAC.C.G.C..CG..TT...T.GC...T.A...CA.GA...
HG03100.1        .AA..TC.AGCTG..G........A..ACC..CC.TCTGA........A......AC.C.G...AC.G.TT..TT.GC...T.A...CA.G..G.
HG02433.0        ....T..GGA.........CGAT.A..A.C..C..TCTG........TA.C...TAC.CTG.C..C...TT...T.GC...T.A...CA.GA...
HG02095.0        ....T..GGG...........A..A..A.C..C..TCTG.........A....A......G....C.G.TT...T.GC...T.A...CA.GA...
HG02095.1        ....T..GGA.........CGAT.A..A.CC.C..T.TG........TA.C...TAC.C.G.C..C...TT...T.GC...T.A...CA.GA..T
```
