# Supplementary figures and images for: Lower promoter activity of the ST8SIA2 gene has been favored in evolving human collective brains
Source: PLoS One. 2021 Dec 16;16(12):e0259897. doi: 10.1371/journal.pone.0259897 (PMC8675693; doi:10.1371/journal.pone.0259897)

**A**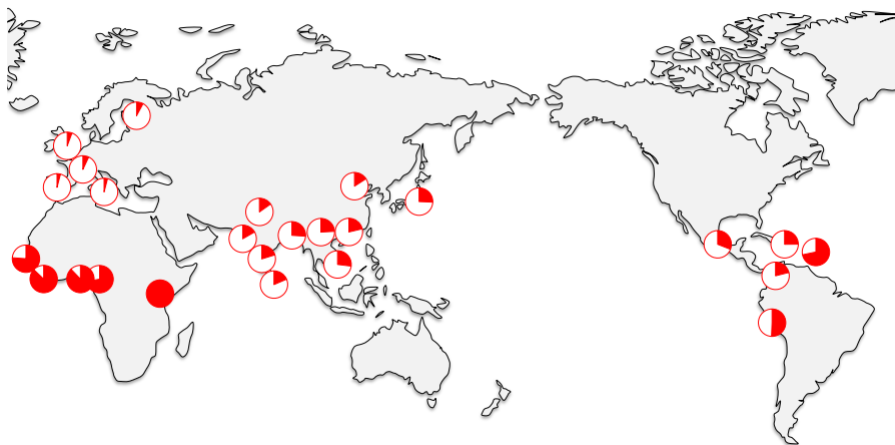**B**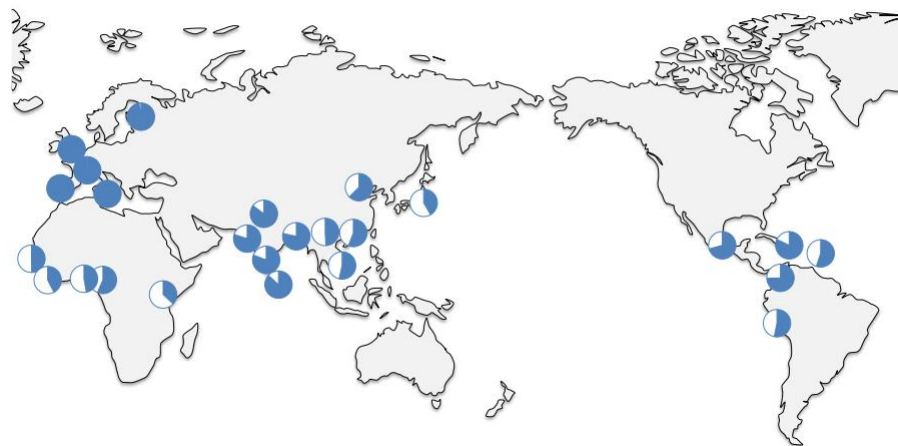**C**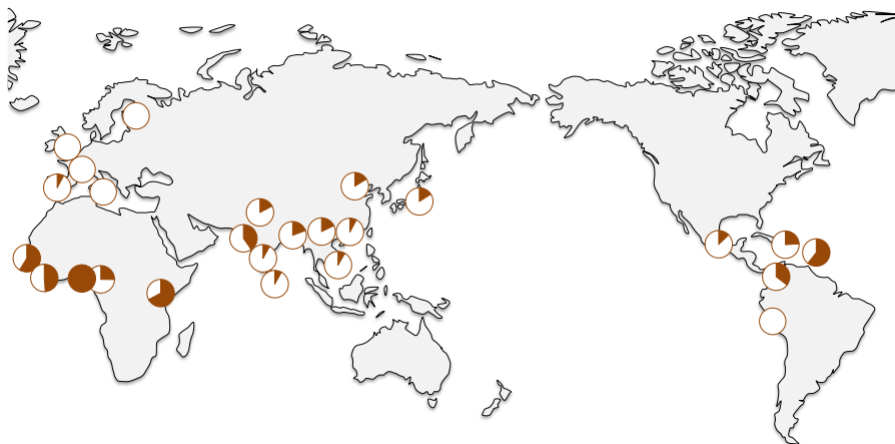**D**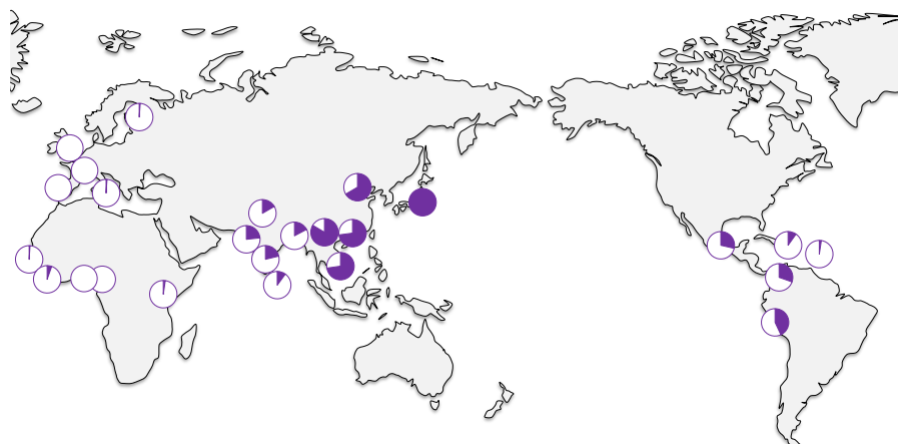

Supplement: S2 Fig — Global distribution of the TGT (A), TCT (B), CGT (C), and CGC (D) sequences. Pie charts represent relative frequencies as compared with the highest frequency among subpopulations [LWK in (A), TSI in (B), YRI in (C), and JPT in (D)]. ASW is not shown because of a lack of information about homelands in Africa. (PDF) [file pone.0259897.s002.pdf]

A

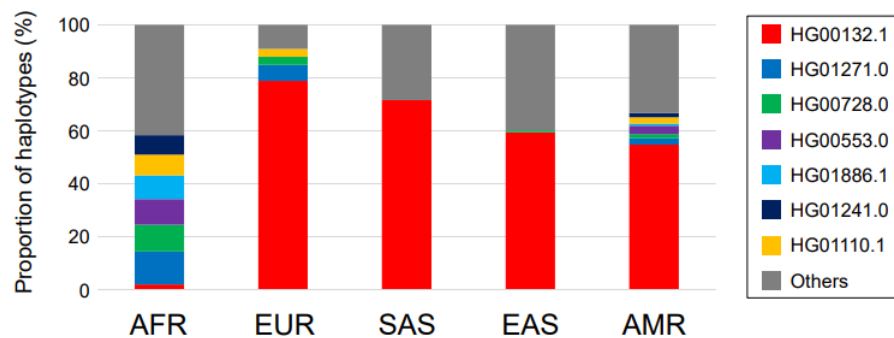

B

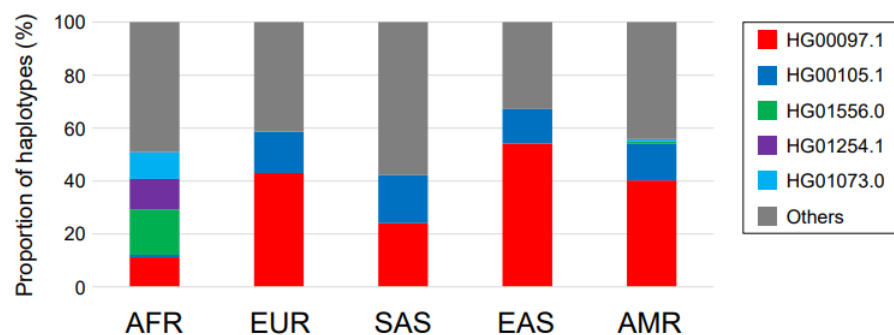

C

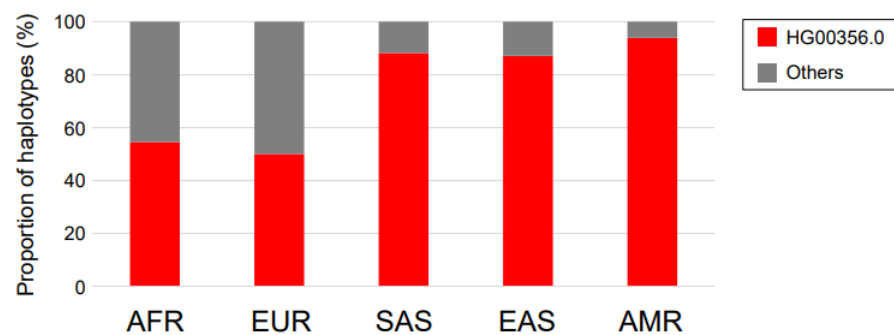

Supplement: S3 Fig — (A) Proportions of major TGT haplotypes (see also Table 1 and S2 Table). (B) Proportions of major TCT haplotypes (see also Table 1 and S3 Table). (C) Proportions of major CGC haplotypes (see also Table 1 and [9]). (PDF) [file pone.0259897.s003.pdf]

**A**

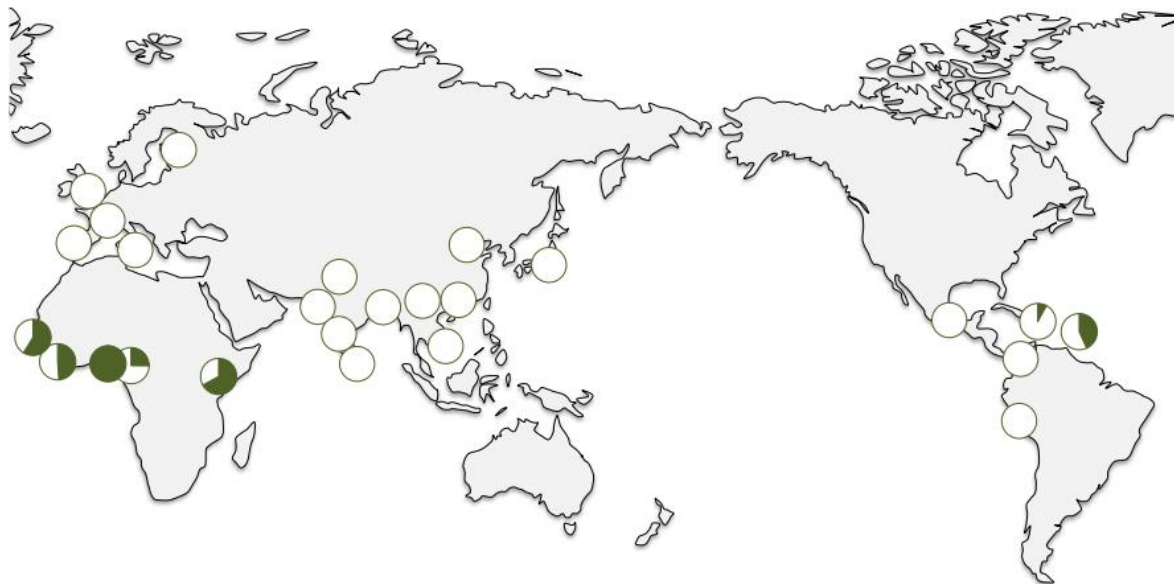

**B**

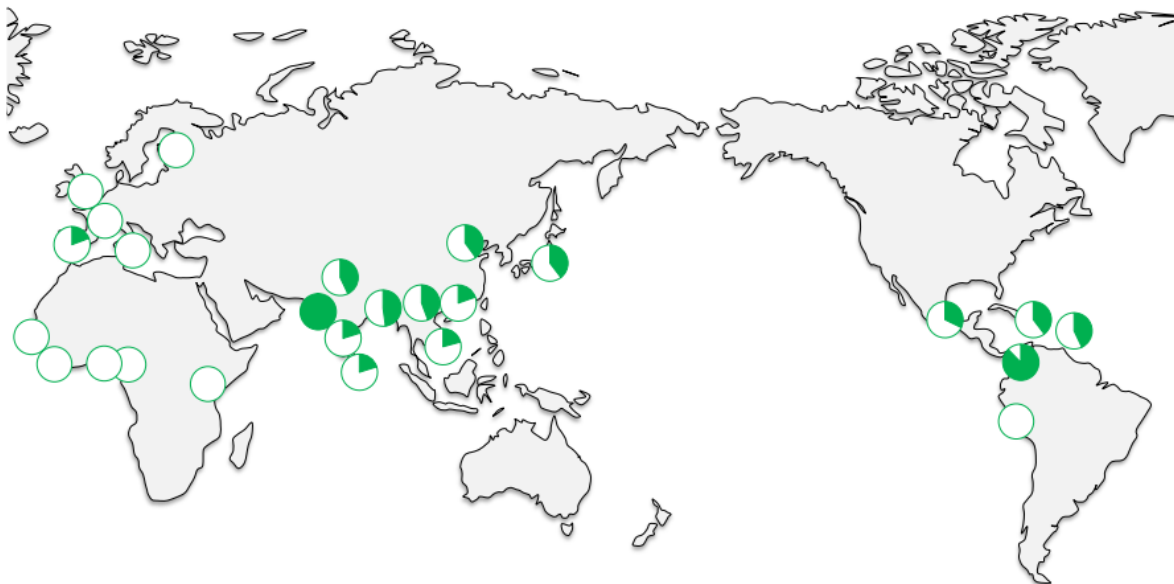

Supplement: S4 Fig — Global distribution of the CGT1 (A) and CGT2 (B) sequences. Pie charts represent relative frequencies as compared with the highest frequency among subpopulations [YRI in (A) and GIH in (B)]. ASW is not shown because of a lack of information about homelands in Africa. (PDF) [file pone.0259897.s004.pdf]

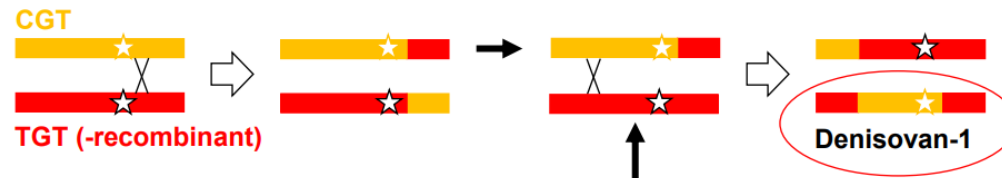

or

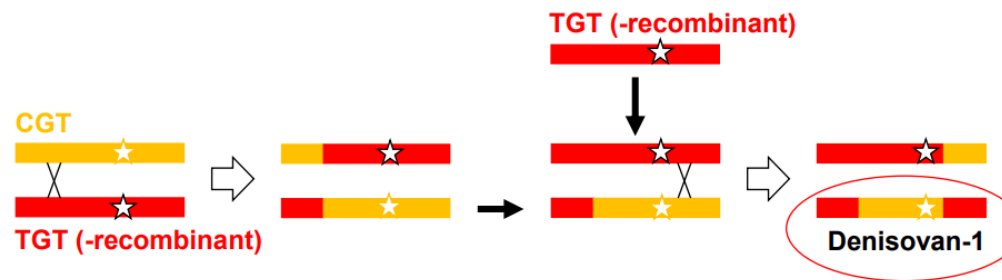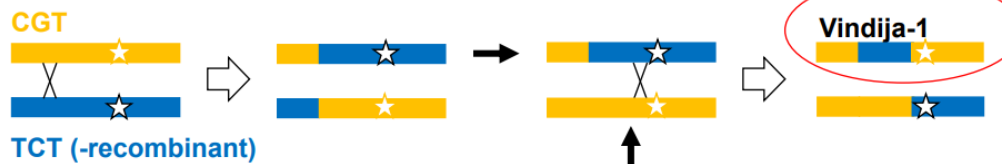

or

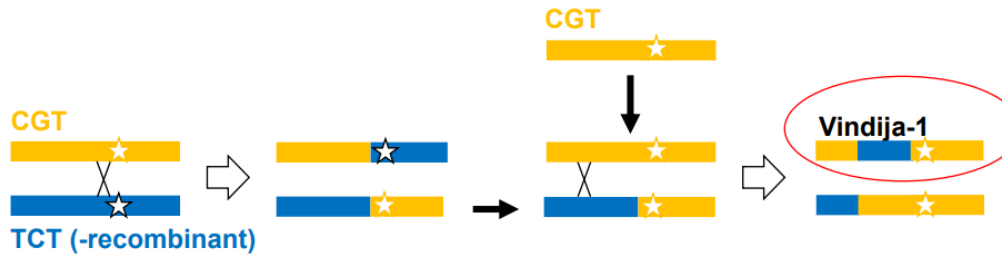

Supplement: S7 Fig — Star represents the position of the three promoter SNPs. (PDF) [file pone.0259897.s007.pdf]

A

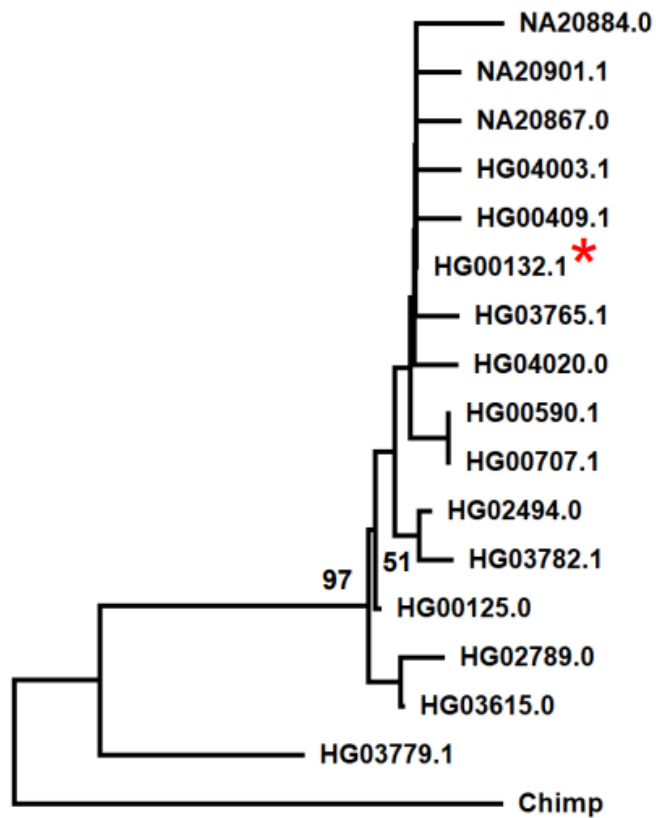

B

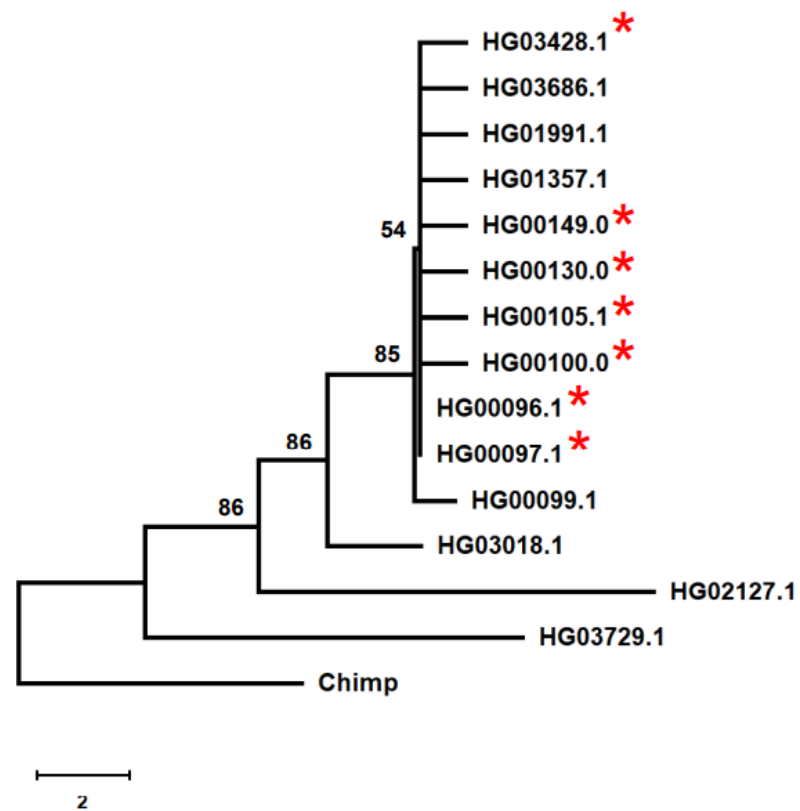

Supplement: S8 Fig — (A) Tree of the TGT haplotypes. (B) Tree of the TCT haplotypes. These haplotypes were selected based on the analyses using site differences (see the “Materials and Methods” section). The phylogenetic trees were constructed by the Neighbor-Joining method with the number of differences. Bootstrap values of more than 50% from 1,000 replications are shown on the tree branches. Red star represents the haplotype found in AFR. (PDF) [file pone.0259897.s008.pdf]

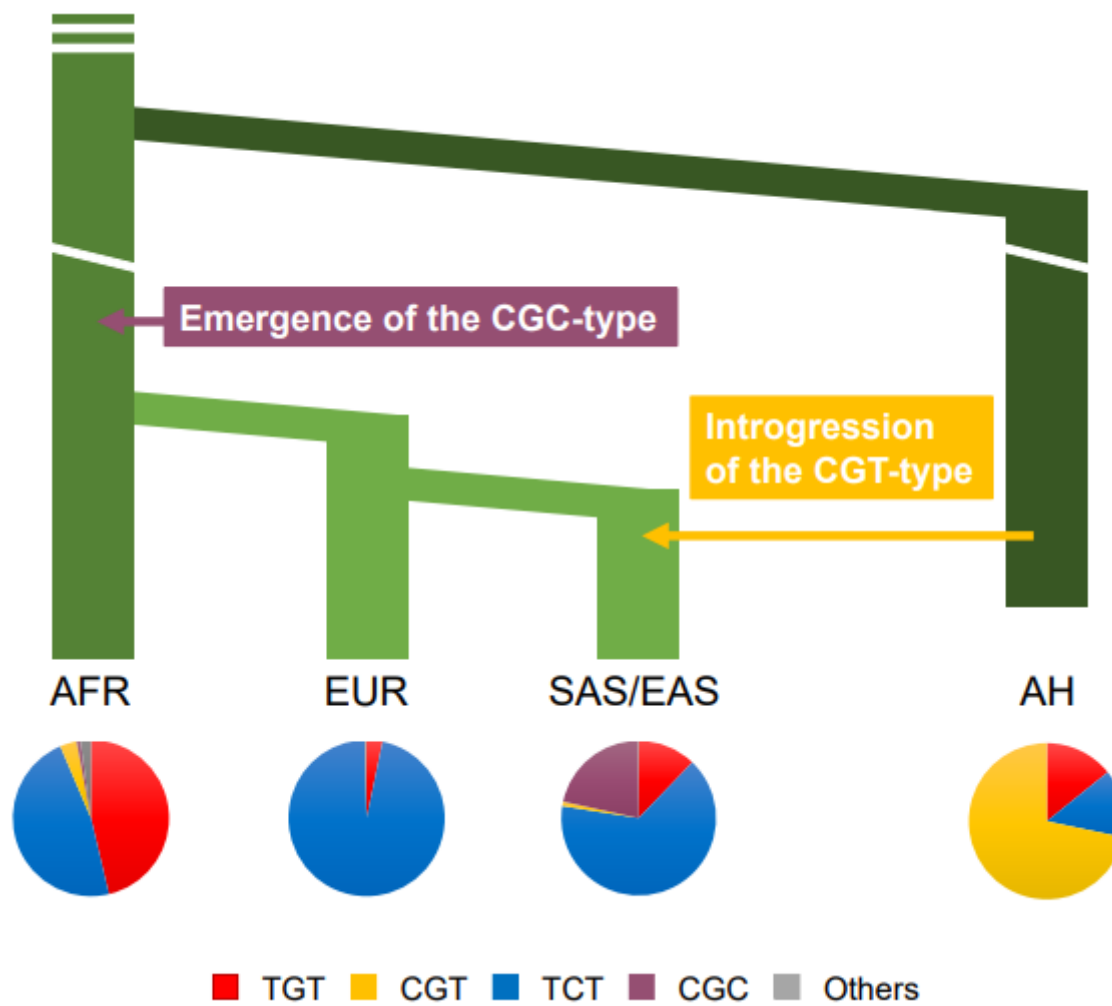

Supplement: S9 Fig — Pie chart of AHs was drawn by promoter-type composition producing the lowest inferred PPA (see S9 Table). (PDF) [file pone.0259897.s009.pdf]

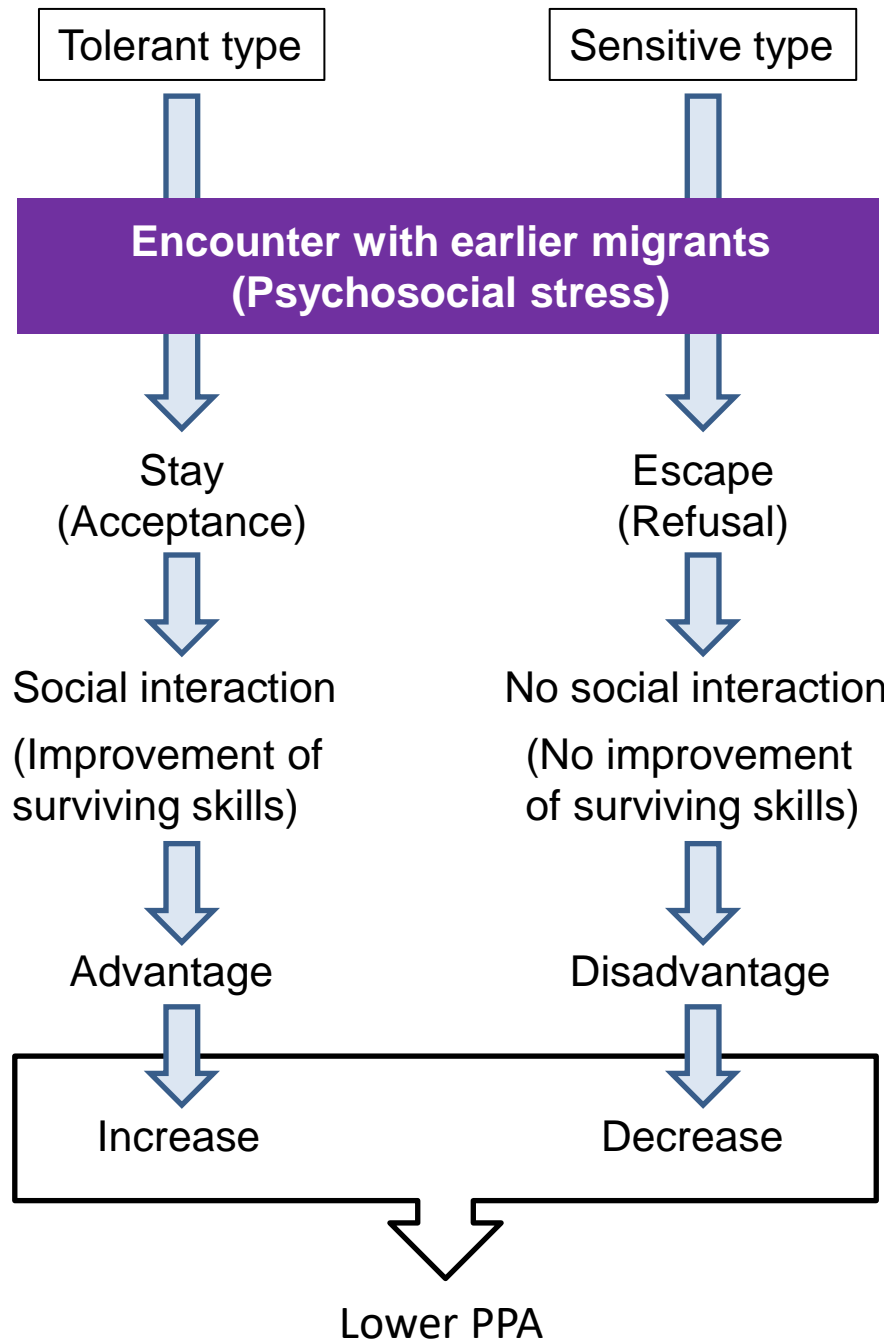

Supplement: S10 Fig — For details of the functional consequence on social interaction, please see our previous report [9]. (PDF) [file pone.0259897.s010.pdf]

**A**

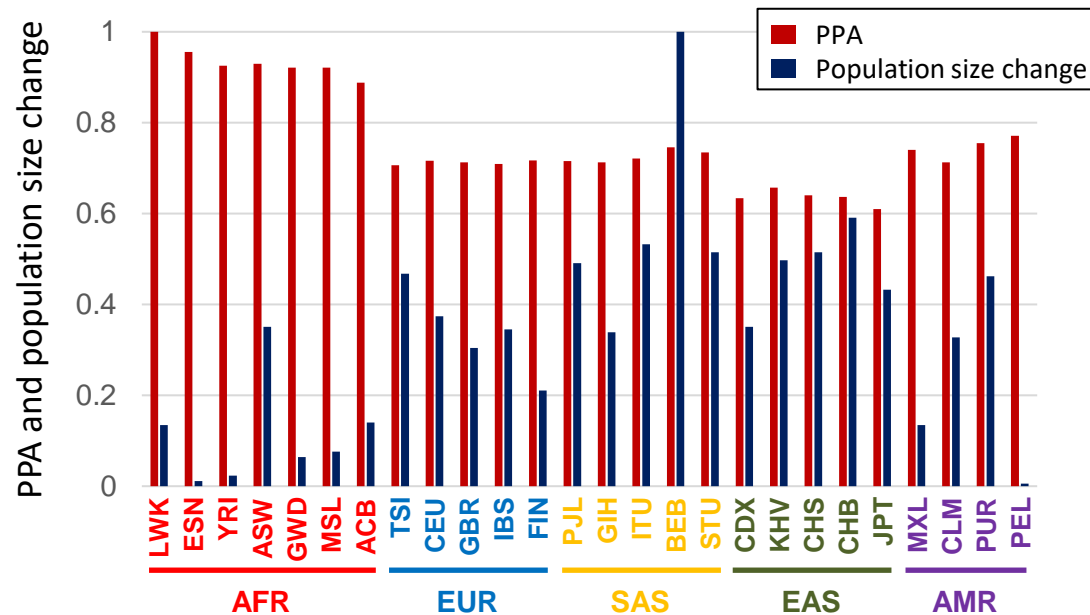

**B**

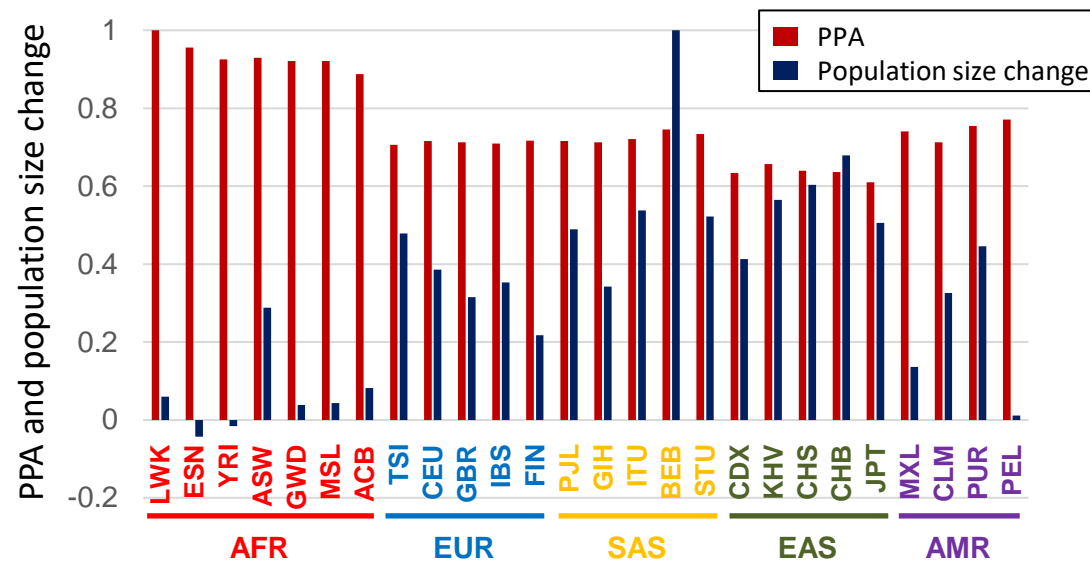

Supplement: S11 Fig — (A) PPAs and effective population size changes obtained roughly from the PSMC smoothed curve [16]. (B) PPAs and effective population size changes obtained roughly from the PSMC unsmoothed curve [16]. As for PPA, relative difference as compared with LWK is shown. As for effective population size change, relative difference as compared with BEB is shown. (PDF) [file pone.0259897.s011.pdf]

## Adaptation to psychosocial stress in the AMH spread

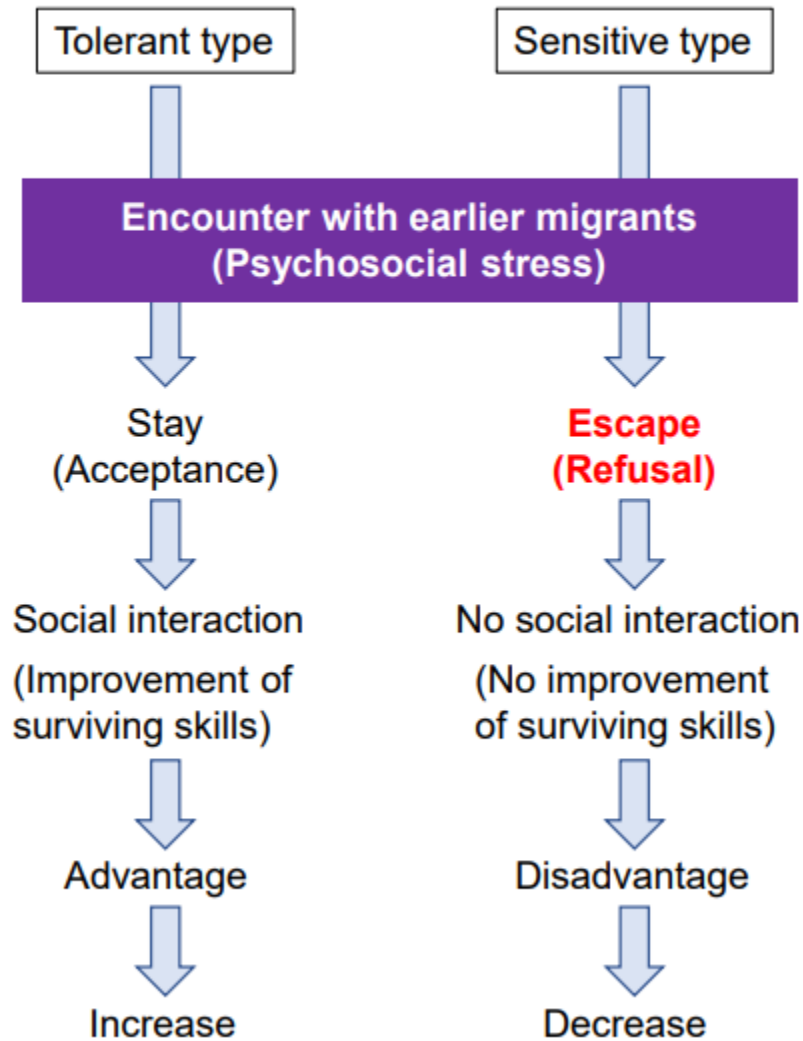

## Schizophrenia onset

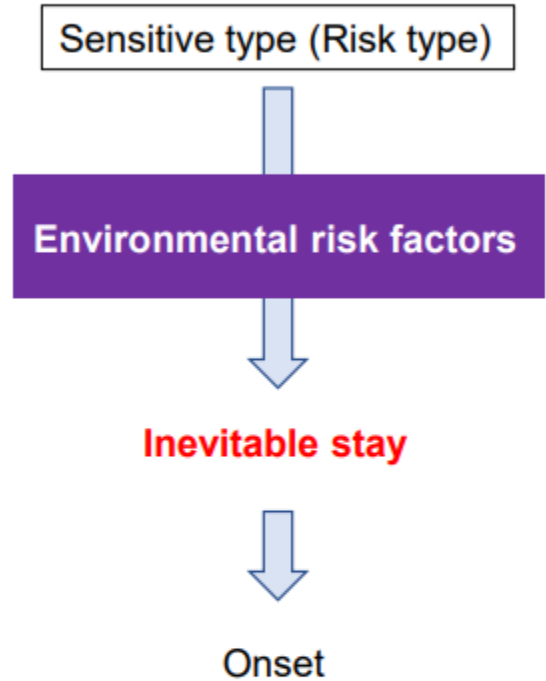

Supplement: S12 Fig — In the onset of schizophrenia, inevitable stay under the environmental risk factors is essential. However, this inevitability of staying is not necessarily assumed in the adaptation to psychosocial stress. (PDF) [file pone.0259897.s012.pdf]
