# Supplementary material for: Lower promoter activity of the ST8SIA2 gene has been favored in evolving human collective brains
Source: PLoS One. 2021 Dec 16;16(12):e0259897. doi: 10.1371/journal.pone.0259897 (PMC8675693; doi:10.1371/journal.pone.0259897)
Supplement: S6 Fig — Except for rs144258052 (0.34% in total; AH-SNP5), SNPs with minor allele frequency of 0.5% or over in the total AMH population are shown. Open boxes represent AH-SNP sites. As for the non-CGT-types, all AFR haplotypes are represented. The three promoter SNPs are represented by asterisks. Longest identical tracts between AH sequences and non-CGT haplotypes are highlighted in blue (Denisovan-1) and green (Vindija-1), and those between CGT1 haplotypes and non-CGT haplotypes are highlighted in orange and yellow. The positions of six SNPs used for the estimation of variant age are indicated in red (see S1 Fig). AH-SNP1 was not used for variant age estimation because the C allele is shared with a TCT haplotype found only in EUR (HG00145.0; two sequences; S3 Table). It is considered that HG00145.0 containing the C allele at AH-SNP1 is a recombinant with a CGT2 haplotype (HG03667.1) found in EUR because of sequence identity in the region surrounding AH-SNP1 between them (S13 Fig). (PDF) [file pone.0259897.s006.pdf]

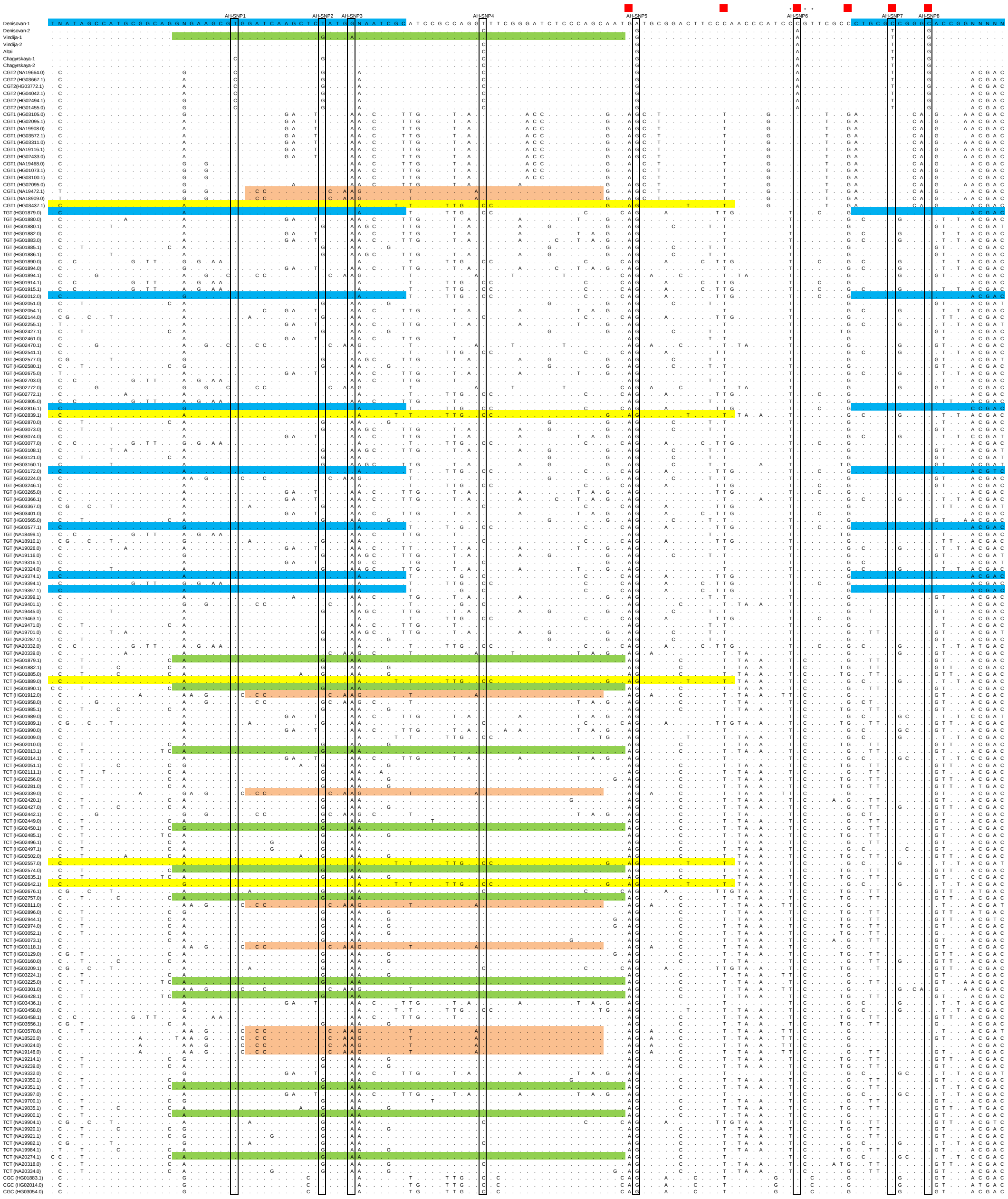
AH-SNP1
AH-SNP2
AH-SNP3
AH-SNP4
AH-SNP5
AH-SNP6
AH-SNP7
AH-SNP8
Denisovan-1
Denisovan-2
Vindija-1
Vindija-2
Altai
Chagyrskaya-1
Chagyrskaya-2
