# Supplementary material for: Lower promoter activity of the ST8SIA2 gene has been favored in evolving human collective brains
Source: PLoS One. 2021 Dec 16;16(12):e0259897. doi: 10.1371/journal.pone.0259897 (PMC8675693; doi:10.1371/journal.pone.0259897)
Supplement: S13 Fig — Except for rs144258052 (0.34% in total; AH-SNP5), SNPs with minor allele frequency of 0.5% or over in the total AMH population are shown. Open boxes represent AH-SNP sites. The three promoter SNPs are represented by asterisks. Longest identical tract surrounding AH-SNP1 between a CGT2 sequence (HG03667.1) and an EUR-unique TCT haplotype (HG00145.0) is highlighted in purple. This sequence identity suggests that HG00145.0 containing the C allele at AH-SNP1 is a recombinant with a CGT2 haplotype (HG03667.1) found in EUR. (PDF) [file pone.0259897.s013.pdf]

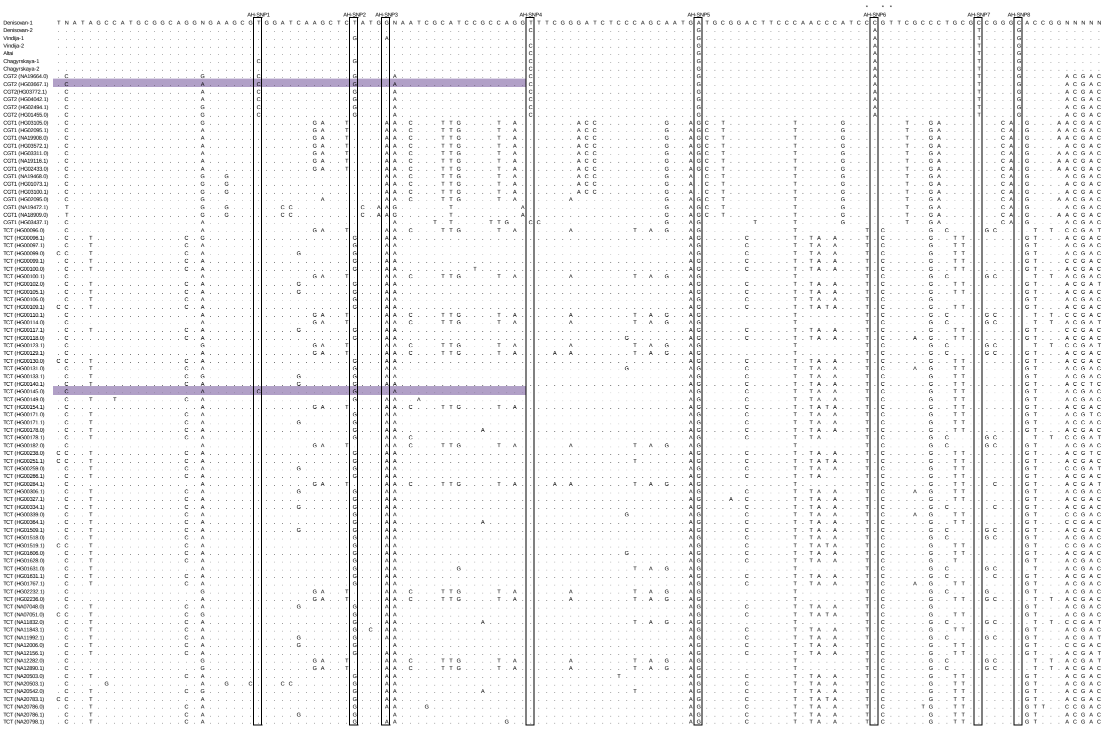
AH-SNP1
AH-SNP2
AH-SNP3
AH-SNP4
AH-SNP5
AH-SNP6
AH-SNP7
AH-SNP8
