# Supplementary material for: Lower promoter activity of the ST8SIA2 gene has been favored in evolving human collective brains
Source: PLoS One. 2021 Dec 16;16(12):e0259897. doi: 10.1371/journal.pone.0259897 (PMC8675693; doi:10.1371/journal.pone.0259897)
Supplement: S1 Table — (PDF) [file pone.0259897.s014.pdf]

S1 Table. Distribution of promoter types

| Promoter type | AFR | EUR | SAS | EAS | AMR | Total |
|---|---|---|---|---|---|---|
| TGT | 612 | 33 | 109 | 133 | 126 | 1013 |
| TCT | 625 | 969 | 782 | 515 | 478 | 3369 |
| CGT | 46 | 1 | 11 | 8 | 8 | 74 |
| CGC | 11 | 2 | 76 | 349 | 82 | 520 |
| Others | 28 | 1 | 0 | 3 | 0 | 32 |
| Total | 1322 | 1006 | 978 | 1008 | 694 | 5008 |
