# Supplementary material for: Lower promoter activity of the ST8SIA2 gene has been favored in evolving human collective brains
Source: PLoS One. 2021 Dec 16;16(12):e0259897. doi: 10.1371/journal.pone.0259897 (PMC8675693; doi:10.1371/journal.pone.0259897)
Supplement: S2 Table — (PDF) [file pone.0259897.s015.pdf]

S2 Table. Distribution of the TGT haplotypes*

	AFR							EUR					SAS					EAS					AMR				
Haplotype	ACB	ASW	ESN	GWD	LWK	MSL	YRI	CEU	FIN	GBR	IBS	TSI	BEB	GIH	ITU	PJL	STU	CHB	CHS	CDX	JPT	KHV	CLM	MXL	PEL	PUR	Total
HG00132.1	1	5		2		2	2	7	9	4	3	3	17	14	18	12	17	11	18	15	20	15	10	14	36	9	264
HG01271.0	12	4	13	11	17	7	12				1	1											3				81
HG00728.0	8	4	9	13	12	8	8				1								1				1			1	66
HG00553.0	10	8	7	6	15	8	5																	1		3	63
HG01886.1	6	7	6	4	5	11	16																	1			56
HG01241.0	1	3	7	9	7	10	11					1												2		1	52
HG01110.1	7	2	5	7	7	12	5																			2	47
HG01082.1	6	3	4	6	4	3	9																			2	37
HG01171.1	3	1	2	11	11	2	4																			1	35
HG00409.1													2					2	3	3	6	8		1	6	2	33
HG01095.0	4	3	5	4	8		4																1			2	31
HG01494.0	3	1	3	4	1	3	5																1				21
HG00707.1														1	1			1	1	1	3	1	2	3	5		19
HG00590.1																	2	4	1	3	2	4	1				17
HG02051.0	4	2	1	4	1	1	1																	1			15
HG01250.1	2	1	2	1	4		3																1				14
HG00422.0																		1	2	4		2	1		2	1	13
HG01894.0	4	1	3	1			3																				12
HG01437.1	1		2	3	2	1	1																1				11
HG00125.0										1			3	1	2	1	1										9
HG01894.1	3			1		4																					8
HG01440.0			2	1	1	1		1															1				7
HG00637.1			2			1	1																			1	5
HG01077.0				2		2																				1	5
HG03074.0		2	1		1	1																					5
NA19026.0					5																						5
NA19374.1					5																						5
HG02577.0	1	1				1	1																				4
HG02772.0				2		2																					4
HG01890.0	1		1				1																				3
HG02789.0													2			1											3
HG03121.0			2			1																					3
NA19401.1		1			2																						3
HG00236.1										1															1		2
HG01067.1																										2	2
HG02054.1	1					1																					2
HG02675.0				1			1																				2
HG02772.1				2																							2
HG03265.0			2																								2
HG03615.0													1				1										2
NA19399.1					2																						2
NA20867.0														2													2
HG01092.1																										1	1
HG01170.0																										1	1
HG01871.0																						1					1
HG02427.1	1																										1
HG02494.0																1											1
HG02580.1	1																										1
HG02660.0																1											1
HG02805.0				1																							1
HG02816.1				1																							1
HG02839.1				1																							1
HG02870.0				1																							1
HG03073.0						1																					1
HG03160.1			1																								1
HG03172.0			1																								1
HG03224.0						1																					1
HG03246.0				1																							1
HG03366.1			1																								1
HG03367.0			1																								1
HG03401.0						1																					1
HG03577.1						1																					1
HG03765.1																1											1
HG03779.1															1												1
HG03782.1															1												1
HG03809.0													1														1
HG04003.1																	1										1
HG04020.0															1												1
NA18499.1							1																				1
NA18910.1							1																				1
NA19116.0							1																				1
NA19316.1					1																						1
NA19324.0					1																						1
NA19445.0					1																						1
NA19463.1					1																						1
NA19471.0					1																						1
NA19701.0		1																									1
NA20287.1		1																									1
NA20339.0		1																									1
NA20884.0														1													1
NA20901.1														1													1
Total	80	52	83	100	115	86	96	8	9	6	5	5	26	20	24	17	22	19	26	26	31	31	23	23	50	30	1013

* SNPs with minor allele frequency of 1% or over in total AMH population were used.
