# Supplementary material for: Lower promoter activity of the ST8SIA2 gene has been favored in evolving human collective brains
Source: PLoS One. 2021 Dec 16;16(12):e0259897. doi: 10.1371/journal.pone.0259897 (PMC8675693; doi:10.1371/journal.pone.0259897)
Supplement: S5 Table — (PDF) [file pone.0259897.s018.pdf]

S5 Table. Matrix of site differences among the CGT haplotypes

	HG03105.0	HG02095.1	NA19908.0	HG03572.1	HG03311.0	NA19116.1	HG02433.0	NA19468.0	HG01073.1	HG03100.1	HG02095.0	NA19472.1	NA18909.0	HG03437.1	NA19664.0	HG03667.1	HG03772.1	HG04042.1	HG02494.1	HG01455.0
HG03105.0																				
HG02095.1	1																			
NA19908.0	1	0																		
HG03572.1	1	0	0																	
HG03311.0	1	0	0	0																
NA19116.1	1	0	0	0	0															
HG02433.0	1	0	0	0	0	0														
NA19468.0	4	5	5	5	5	5	5													
HG01073.11	4	5	5	5	5	5	5	0												
HG03100.	4	5	5	5	5	5	5	0	0											
HG02095.0	3	4	4	4	4	4	4	3	3	3										
NA19472.1	15	16	16	16	16	16	16	11	11	11	14									
NA18909.0	15	16	16	16	16	16	16	11	11	11	14	0								
HG03437.1	15	14	14	14	14	14	14	13	13	13	14	16	16							
NA19664.0	14	15	15	15	15	15	15	12	12	12	13	13	13	11						
HG03667.1	15	14	14	14	14	14	14	13	13	13	14	14	14	10	1					
HG03772.1	15	14	14	14	14	14	14	13	13	13	14	14	14	10	1	0				
HG04042.1	15	14	14	14	14	14	14	13	13	13	14	14	14	10	1	0	0			
HG02494.1	14	15	15	15	15	15	15	12	12	12	13	13	13	11	0	1	1	1		
HG01455.0	14	15	15	15	15	15	15	12	12	12	13	13	13	11	0	1	1	1	0	
