# Supplementary material for: Lower promoter activity of the ST8SIA2 gene has been favored in evolving human collective brains
Source: PLoS One. 2021 Dec 16;16(12):e0259897. doi: 10.1371/journal.pone.0259897 (PMC8675693; doi:10.1371/journal.pone.0259897)
Supplement: S8 Table — (PDF) [file pone.0259897.s021.pdf]

S8 Table. Matrix of site differences among the CGC haplotypes from SAS

	HG03817.1	HG00356.0	HG02069.0	HG03809.1	HG03870.1	HG02490.0	HG03868.0
HG03817.1							
HG00356.0	1						
HG02069.0	3	2					
HG03809.1	23	22	24				
HG03870.1	1	0	2	22			
HG02490.0	1	0	2	22	0		
HG03868.0	1	0	2	22	0	0	
