# Supplementary material for: Lower promoter activity of the ST8SIA2 gene has been favored in evolving human collective brains
Source: PLoS One. 2021 Dec 16;16(12):e0259897. doi: 10.1371/journal.pone.0259897 (PMC8675693; doi:10.1371/journal.pone.0259897)
Supplement: S9 Table — (PDF) [file pone.0259897.s022.pdf]

S9 Table. Inference of promoter type composition and PPA of AH population.

Promoter type frequency			Genotype frequency						PPA	Difference of PPA relative to AFR	Difference of PPA relative to LWK
CGT	TGT	TCT	CGT/CGT	CGT/TGT	CGT/TCT	TGT/TGT	TCT/TCT	TGT/TCT			
0.717	0.142	0.141	0.514	0.204	0.202	0.020	0.020	0.040	2.891	1.010	0.944
0.717	0.152	0.131	0.514	0.218	0.188	0.023	0.017	0.040	2.906	1.016	0.949
0.717	0.162	0.121	0.514	0.232	0.174	0.026	0.015	0.039	2.921	1.021	0.954
0.717	0.172	0.111	0.514	0.247	0.159	0.030	0.012	0.038	2.936	1.026	0.959
0.717	0.182	0.101	0.514	0.261	0.145	0.033	0.010	0.037	2.951	1.031	0.963
0.717	0.192	0.091	0.514	0.275	0.130	0.037	0.008	0.035	2.966	1.037	0.968
0.717	0.202	0.081	0.514	0.290	0.116	0.041	0.007	0.033	2.981	1.042	0.973
0.717	0.212	0.071	0.514	0.304	0.102	0.045	0.005	0.030	2.996	1.047	0.978
0.717	0.222	0.061	0.514	0.318	0.087	0.049	0.004	0.027	3.011	1.052	0.983
0.717	0.232	0.051	0.514	0.333	0.073	0.054	0.003	0.024	3.026	1.058	0.988
0.717	0.242	0.041	0.514	0.347	0.059	0.059	0.002	0.020	3.041	1.063	0.993
0.717	0.252	0.031	0.514	0.361	0.044	0.064	0.001	0.016	3.056	1.068	0.998
0.717	0.262	0.021	0.514	0.376	0.030	0.069	0.000	0.011	3.071	1.073	1.003
0.717	0.272	0.011	0.514	0.390	0.016	0.074	0.000	0.006	3.086	1.079	1.008
0.72	0.15	0.13	0.518	0.216	0.187	0.023	0.017	0.039	2.905	1.015	0.948
0.72	0.16	0.12	0.518	0.230	0.173	0.026	0.014	0.038	2.920	1.021	0.953
0.72	0.17	0.11	0.518	0.245	0.158	0.029	0.012	0.037	2.935	1.026	0.958
0.72	0.18	0.1	0.518	0.259	0.144	0.032	0.010	0.036	2.950	1.031	0.963
0.72	0.19	0.09	0.518	0.274	0.130	0.036	0.008	0.034	2.965	1.036	0.968
0.72	0.2	0.08	0.518	0.288	0.115	0.040	0.006	0.032	2.980	1.042	0.973
0.72	0.21	0.07	0.518	0.302	0.101	0.044	0.005	0.029	2.995	1.047	0.978
0.72	0.22	0.06	0.518	0.317	0.086	0.048	0.004	0.026	3.010	1.052	0.983
0.72	0.23	0.05	0.518	0.331	0.072	0.053	0.003	0.023	3.025	1.057	0.988
0.72	0.24	0.04	0.518	0.346	0.058	0.058	0.002	0.019	3.041	1.063	0.993
0.72	0.25	0.03	0.518	0.360	0.043	0.063	0.001	0.015	3.056	1.068	0.998
0.72	0.26	0.02	0.518	0.374	0.029	0.068	0.000	0.010	3.071	1.073	1.002
0.72	0.27	0.01	0.518	0.389	0.014	0.073	0.000	0.005	3.086	1.078	1.007
0.73	0.14	0.13	0.533	0.204	0.190	0.020	0.017	0.036	2.898	1.013	0.946
0.73	0.15	0.12	0.533	0.219	0.175	0.023	0.014	0.036	2.913	1.018	0.951
0.73	0.16	0.11	0.533	0.234	0.161	0.026	0.012	0.035	2.928	1.023	0.956
0.73	0.17	0.1	0.533	0.248	0.146	0.029	0.010	0.034	2.943	1.028	0.961
0.73	0.18	0.09	0.533	0.263	0.131	0.032	0.008	0.032	2.958	1.034	0.966
0.73	0.19	0.08	0.533	0.277	0.117	0.036	0.006	0.030	2.973	1.039	0.971
0.73	0.2	0.07	0.533	0.292	0.102	0.040	0.005	0.028	2.988	1.044	0.975
0.73	0.21	0.06	0.533	0.307	0.088	0.044	0.004	0.025	3.003	1.049	0.980
0.73	0.22	0.05	0.533	0.321	0.073	0.048	0.003	0.022	3.018	1.055	0.985
0.73	0.23	0.04	0.533	0.336	0.058	0.053	0.002	0.018	3.033	1.060	0.990
0.73	0.24	0.03	0.533	0.350	0.044	0.058	0.001	0.014	3.048	1.065	0.995
0.73	0.25	0.02	0.533	0.365	0.029	0.063	0.000	0.010	3.063	1.070	1.000
0.73	0.26	0.01	0.533	0.380	0.015	0.068	0.000	0.005	3.078	1.076	1.005
0.74	0.14	0.12	0.548	0.207	0.178	0.020	0.014	0.034	2.905	1.015	0.948
0.74	0.15	0.11	0.548	0.222	0.163	0.023	0.012	0.033	2.920	1.021	0.953
0.74	0.16	0.1	0.548	0.237	0.148	0.026	0.010	0.032	2.935	1.026	0.958
0.74	0.17	0.09	0.548	0.252	0.133	0.029	0.008	0.031	2.950	1.031	0.963
0.74	0.18	0.08	0.548	0.266	0.118	0.032	0.006	0.029	2.966	1.036	0.968
0.74	0.19	0.07	0.548	0.281	0.104	0.036	0.005	0.027	2.981	1.042	0.973
0.74	0.2	0.06	0.548	0.296	0.089	0.040	0.004	0.024	2.996	1.047	0.978
0.74	0.21	0.05	0.548	0.311	0.074	0.044	0.003	0.021	3.011	1.052	0.983
0.74	0.22	0.04	0.548	0.326	0.059	0.048	0.002	0.018	3.026	1.057	0.988
0.74	0.23	0.03	0.548	0.340	0.044	0.053	0.001	0.014	3.041	1.063	0.993
0.74	0.24	0.02	0.548	0.355	0.030	0.058	0.000	0.010	3.056	1.068	0.998
0.74	0.25	0.01	0.548	0.370	0.015	0.063	0.000	0.005	3.071	1.073	1.002
0.75	0.13	0.12	0.563	0.195	0.180	0.017	0.014	0.031	2.898	1.013	0.946
0.75	0.14	0.11	0.563	0.210	0.165	0.020	0.012	0.031	2.913	1.018	0.951
0.75	0.15	0.1	0.563	0.225	0.150	0.023	0.010	0.030	2.928	1.023	0.956
0.75	0.16	0.09	0.563	0.240	0.135	0.026	0.008	0.029	2.943	1.028	0.961
0.75	0.17	0.08	0.563	0.255	0.120	0.029	0.006	0.027	2.958	1.034	0.966
0.75	0.18	0.07	0.563	0.270	0.105	0.032	0.005	0.025	2.973	1.039	0.971
0.75	0.19	0.06	0.563	0.285	0.090	0.036	0.004	0.023	2.988	1.044	0.976
0.75	0.2	0.05	0.563	0.300	0.075	0.040	0.003	0.020	3.003	1.049	0.980
0.75	0.21	0.04	0.563	0.315	0.060	0.044	0.002	0.017	3.018	1.055	0.985
0.75	0.22	0.03	0.563	0.330	0.045	0.048	0.001	0.013	3.033	1.060	0.990
0.75	0.23	0.02	0.563	0.345	0.030	0.053	0.000	0.009	3.048	1.065	0.995
0.75	0.24	0.01	0.563	0.360	0.015	0.058	0.000	0.005	3.063	1.071	1.000
0.76	0.13	0.11	0.578	0.198	0.167	0.017	0.012	0.029	2.906	1.015	0.949
0.76	0.14	0.1	0.578	0.213	0.152	0.020	0.010	0.028	2.921	1.021	0.953
0.76	0.15	0.09	0.578	0.228	0.137	0.023	0.008	0.027	2.936	1.026	0.958
0.76	0.16	0.08	0.578	0.243	0.122	0.026	0.006	0.026	2.951	1.031	0.963
0.76	0.17	0.07	0.578	0.258	0.106	0.029	0.005	0.024	2.966	1.036	0.968
0.76	0.18	0.06	0.578	0.274	0.091	0.032	0.004	0.022	2.981	1.042	0.973
0.76	0.19	0.05	0.578	0.289	0.076	0.036	0.003	0.019	2.996	1.047	0.978
0.76	0.2	0.04	0.578	0.304	0.061	0.040	0.002	0.016	3.011	1.052	0.983
0.76	0.21	0.03	0.578	0.319	0.046	0.044	0.001	0.013	3.026	1.057	0.988
0.76	0.22	0.02	0.578	0.334	0.030	0.048	0.000	0.009	3.041	1.063	0.993
0.76	0.23	0.01	0.578	0.350	0.015	0.053	0.000	0.005	3.056	1.068	0.998
0.77	0.12	0.11	0.593	0.185	0.169	0.014	0.012	0.026	2.898	1.013	0.946
0.77	0.13	0.1	0.593	0.200	0.154	0.017	0.010	0.026	2.913	1.018	0.951
0.77	0.14	0.09	0.593	0.216	0.139	0.020	0.008	0.025	2.928	1.023	0.956
0.77	0.15	0.08	0.593	0.231	0.123	0.023	0.006	0.024	2.943	1.029	0.961
0.77	0.16	0.07	0.593	0.246	0.108	0.026	0.005	0.022	2.958	1.034	0.966
0.77	0.17	0.06	0.593	0.262	0.092	0.029	0.004	0.020	2.973	1.039	0.971
0.77	0.18	0.05	0.593	0.277	0.077	0.032	0.003	0.018	2.988	1.044	0.976
0.77	0.19	0.04	0.593	0.293	0.062	0.036	0.002	0.015	3.003	1.050	0.980
0.77	0.2	0.03	0.593	0.308	0.046	0.040	0.001	0.012	3.018	1.055	0.985
0.77	0.21	0.02	0.593	0.323	0.031	0.044	0.000	0.008	3.033	1.060	0.990
0.77	0.22	0.01	0.593	0.339	0.015	0.048	0.000	0.004	3.048	1.065	0.995
0.78	0.12	0.1	0.608	0.187	0.156	0.014	0.010	0.024	2.906	1.015	0.949
0.78	0.13	0.09	0.608	0.203	0.140	0.017	0.008	0.023	2.921	1.021	0.953
0.78	0.14	0.08	0.608	0.218	0.125	0.020	0.006	0.022	2.936	1.026	0.958
0.78	0.15	0.07	0.608	0.234	0.109	0.023	0.005	0.021	2.951	1.031	0.963
0.78	0.16	0.06	0.608	0.250	0.094	0.026	0.004	0.019	2.966	1.036	0.968
0.78	0.17	0.05	0.608	0.265	0.078	0.029	0.003	0.017	2.981	1.042	0.973
0.78	0.18	0.04	0.608	0.281	0.062	0.032	0.002	0.014	2.996	1.047	0.978
0.78	0.19	0.03	0.608	0.296	0.047	0.036	0.001	0.011	3.011	1.052	0.983
0.78	0.2	0.02	0.608	0.312	0.031	0.040	0.000	0.008	3.026	1.057	0.988
0.78	0.21	0.01	0.608	0.328	0.016	0.044	0.000	0.004	3.041	1.063	0.993
0.79	0.11	0.1	0.624	0.174	0.158	0.012	0.010	0.022	2.898	1.013	0.946
0.79	0.12	0.09	0.624	0.190	0.142	0.014	0.008	0.022	2.913	1.018	0.951
0.79	0.13	0.08	0.624	0.205	0.126	0.017	0.006	0.021	2.928	1.023	0.956
0.79	0.14	0.07	0.624	0.221	0.111	0.020	0.005	0.020	2.943	1.029	0.961
0.79	0.15	0.06	0.624	0.237	0.095	0.023	0.004	0.018	2.958	1.034	0.966
0.79	0.16	0.05	0.624	0.253	0.079	0.026	0.003	0.016	2.973	1.039	0.971
0.79	0.17	0.04	0.624	0.269	0.063	0.029	0.002	0.014	2.988	1.044	0.976
0.79	0.18	0.03	0.624	0.284	0.047	0.032	0.001	0.011	3.003	1.050	0.981
0.79	0.19	0.02	0.624	0.300	0.032	0.036	0.000	0.008	3.018	1.055	0.985
0.79	0.2	0.01	0.624	0.316	0.016	0.040	0.000	0.004	3.033	1.060	0.990
0.8	0.11	0.09	0.640	0.176	0.144	0.012	0.008	0.020	2.906	1.015	0.949
0.8	0.12	0.08	0.640	0.192	0.128	0.014	0.006	0.019	2.921	1.021	0.954
0.8	0.13	0.07	0.640	0.208	0.112	0.017	0.005	0.018	2.936	1.026	0.958
0.8	0.14	0.06	0.640	0.224	0.096	0.020	0.004	0.017	2.951	1.031	0.963
0.8	0.15	0.05	0.640	0.240	0.080	0.023	0.003	0.015	2.966	1.036	0.968
0.8	0.16	0.04	0.640	0.256	0.064	0.026	0.002	0.013	2.981	1.042	0.973
0.8	0.17	0.03	0.640	0.272	0.048	0.029	0.001	0.010	2.996	1.047	0.978
0.8	0.18	0.02	0.640	0.288	0.032	0.032	0.000	0.007	3.011	1.052	0.983
0.8	0.19	0.01	0.640	0.304	0.016	0.036	0.000	0.004	3.026	1.057	0.988
0.81	0.1	0.09	0.656	0.162	0.146	0.010	0.008	0.018	2.898	1.013	0.946
0.81	0.11	0.08	0.656	0.178	0.130	0.012	0.006	0.018	2.913	1.018	0.951
0.81	0.12	0.07	0.656	0.194	0.113	0.014	0.005	0.017	2.928	1.023	0.956
0.81	0.13	0.06	0.656	0.211	0.097	0.017	0.004	0.016	2.943	1.029	0.961
0.81	0.14	0.05	0.656	0.227	0.081	0.020	0.003	0.014	2.958	1.034	0.966
0.81	0.15	0.04	0.656	0.243	0.065	0.023	0.002	0.012	2.973	1.039	0.971
0.81	0.16	0.03	0.656	0.259	0.049	0.026	0.001	0.010	2.989	1.044	0.976
0.81	0.17	0.02	0.656	0.275	0.032	0.029	0.000	0.007	3.004	1.050	0.981
0.81	0.18	0.01	0.656	0.292	0.016	0.032	0.000	0.004	3.019	1.055	0.985
0.82	0.1	0.08	0.672	0.164	0.131	0.010	0.006	0.016	2.906	1.016	0.949
0.82	0.11	0.07	0.672	0.180	0.115	0.012	0.005	0.015	2.921	1.021	0.954
0.82	0.12	0.06	0.672	0.197	0.098	0.014	0.004	0.014	2.936	1.026	0.958
0.82	0.13	0.05	0.672	0.213	0.082	0.017	0.003	0.013	2.951	1.031	0.963
0.82	0.14	0.04	0.672	0.230	0.066	0.020	0.002	0.011	2.966	1.037	0.968
0.82	0.15	0.03	0.672	0.246	0.049	0.023	0.001	0.009	2.981	1.042	0.973
0.82	0.16	0.02	0.672	0.262	0.033	0.026	0.000	0.006	2.996	1.047	0.978
0.82	0.17	0.01	0.672	0.279	0.016	0.029	0.000	0.003	3.011	1.052	0.983
0.83	0.09	0.08	0.689	0.149	0.133	0.008	0.006	0.014	2.898	1.013	0.946
0.83	0.1	0.07	0.689	0.166	0.116	0.010	0.005	0.014	2.914	1.018	0.951
0.83	0.11	0.06	0.689	0.183	0.100	0.012	0.004	0.013	2.929	1.023	0.956
0.83	0.12	0.05	0.689	0.199	0.083	0.014	0.003	0.012	2.944	1.029	0.961
0.83	0.13	0.04	0.689	0.216	0.066	0.017	0.002	0.010	2.959	1.034	0.966
0.83	0.14	0.03	0.689	0.232	0.050	0.020	0.001	0.008	2.974	1.039	0.971
0.83	0.15	0.02	0.689	0.249	0.033	0.023	0.000	0.006	2.989	1.044	0.976
0.83	0.16	0.01	0.689	0.266	0.017	0.026	0.000	0.003	3.004	1.050	0.981
0.84	0.09	0.07	0.706	0.151	0.118	0.008	0.005	0.013	2.906	1.016	0.949
0.84	0.1	0.06	0.706	0.168	0.101	0.010	0.004	0.012	2.921	1.021	0.954
0.84	0.11	0.05	0.706	0.185	0.084	0.012	0.003	0.011	2.936	1.026	0.959
0.84	0.12	0.04	0.706	0.202	0.067	0.014	0.002	0.010	2.951	1.031	0.963
0.84	0.13	0.03	0.706	0.218	0.050	0.017	0.001	0.008	2.966	1.037	0.968
0.84	0.14	0.02	0.706	0.235	0.034	0.020	0.000	0.006	2.981	1.042	0.973
0.84	0.15	0.01	0.706	0.252	0.017	0.023	0.000	0.003	2.996	1.047	0.978
0.85	0.08	0.07	0.723	0.136	0.119	0.006	0.005	0.011	2.899	1.013	0.946
0.85	0.09	0.06	0.723	0.153	0.102	0.008	0.004	0.011	2.914	1.018	0.951
0.85	0.1	0.05	0.723	0.170	0.085	0.010	0.003	0.010	2.929	1.023	0.956
0.85	0.11	0.04	0.723	0.187	0.068	0.012	0.002	0.009	2.944	1.029	0.961
0.85	0.12	0.03	0.723	0.204	0.051	0.014	0.001	0.007	2.959	1.034	0.966
0.85	0.13	0.02	0.723	0.221	0.034	0.017	0.000	0.005	2.974	1.039	0.971
0.85	0.14	0.01	0.723	0.238	0.017	0.020	0.000	0.003	2.989	1.044	0.976
0.86	0.08	0.06	0.740	0.138	0.103	0.006	0.004	0.010	2.906	1.016	0.949
0.86	0.09	0.05	0.740	0.155	0.086	0.008	0.003	0.009	2.921	1.021	0.954
0.86	0.1	0.04	0.740	0.172	0.069	0.010	0.002	0.008	2.936	1.026	0.959
0.86	0.11	0.03	0.740	0.189	0.052	0.012	0.001	0.007	2.951	1.031	0.963
0.86	0.12	0.02	0.740	0.206	0.034	0.014	0.000	0.005	2.966	1.037	0.968
0.86	0.13	0.01	0.740	0.224	0.017	0.017	0.000	0.003	2.981	1.042	0.973
0.87	0.07	0.06	0.757	0.122	0.104	0.005	0.004	0.008	2.899	1.013	0.946
0.87	0.08	0.05	0.757	0.139	0.087	0.006	0.003	0.008	2.914	1.018	0.951
0.87	0.09	0.04	0.757	0.157	0.070	0.008	0.002	0.007	2.929	1.024	0.956
0.87	0.1	0.03	0.757	0.174	0.052	0.010	0.001	0.006	2.944	1.029	0.961
0.87	0.11	0.02	0.757	0.191	0.035	0.012	0.000	0.004	2.959	1.034	0.966
0.87	0.12	0.01	0.757	0.209	0.017	0.014	0.000	0.002	2.974	1.039	0.971
0.88	0.07	0.05	0.774	0.123	0.088	0.005	0.003	0.007	2.906	1.016	0.949
0.88	0.08	0.04	0.774	0.141	0.070	0.006	0.002	0.006	2.921	1.021	0.954
0.88	0.09	0.03	0.774	0.158	0.053	0.008	0.001	0.005	2.936	1.026	0.959
0.88	0.1	0.02	0.774	0.176	0.035	0.010	0.000	0.004	2.951	1.031	0.964
0.88	0.11	0.01	0.774	0.194	0.018	0.012	0.000	0.002	2.966	1.037	0.968
0.89	0.06	0.05	0.792	0.107	0.089	0.004	0.003	0.006	2.899	1.013	0.946
0.89	0.07	0.04	0.792	0.125	0.071	0.005	0.002	0.006	2.914	1.018	0.951
0.89	0.08	0.03	0.792	0.142	0.053	0.006	0.001	0.005	2.929	1.024	0.956
0.89	0.09	0.02	0.792	0.160	0.036	0.008	0.000	0.004	2.944	1.029	0.961
0.89	0.1	0.01	0.792	0.178	0.018	0.010	0.000	0.002	2.959	1.034	0.966
0.9	0.06	0.04	0.810	0.108	0.072	0.004	0.002	0.005	2.906	1.016	0.949
0.9	0.07	0.03	0.810	0.126	0.054	0.005	0.001	0.004	2.921	1.021	0.954
0.9	0.08	0.02	0.810	0.144	0.036	0.006	0.000	0.003	2.937	1.026	0.959
0.9	0.09	0.01	0.810	0.162	0.018	0.008	0.000	0.002	2.952	1.031	0.964
0.91	0.05	0.04	0.828	0.091	0.073	0.003	0.002	0.004	2.899	1.013	0.946
0.91	0.06	0.03	0.828	0.109	0.055	0.004	0.001	0.004	2.914	1.018	0.951
0.91	0.07	0.02	0.828	0.127	0.036	0.005	0.000	0.003	2.929	1.024	0.956
0.91	0.08	0.01	0.828	0.146	0.018	0.006	0.000	0.002	2.944	1.029	0.961
0.92	0.05	0.03	0.846	0.092	0.055	0.003	0.001	0.003	2.907	1.016	0.949
0.92	0.06	0.02	0.846	0.110	0.037	0.004	0.000	0.002	2.922	1.021	0.954
0.92	0.07	0.01	0.846	0.129	0.018	0.005	0.000	0.001	2.937	1.026	0.959
0.93	0.04	0.03	0.865	0.074	0.056	0.002	0.001	0.002	2.899	1.013	0.946
0.93	0.05	0.02	0.865	0.093	0.037	0.003	0.000	0.002	2.914	1.018	0.951
0.93	0.06	0.01	0.865	0.112	0.019	0.004	0.000	0.001	2.929	1.024	0.956
0.94	0.04	0.02	0.884	0.075	0.038	0.002	0.000	0.002	2.907	1.016	0.949
0.94	0.05	0.01	0.884	0.094	0.019	0.003	0.000	0.001	2.922	1.021	0.954
0.95	0.03	0.02	0.903	0.057	0.038	0.001	0.000	0.001	2.899	1.013	0.947
0.95	0.04	0.01	0.903	0.076	0.019	0.002	0.000	0.001	2.914	1.018	0.951
0.96	0.03	0.01	0.922	0.058	0.019	0.001	0.000	0.001	2.907	1.016	0.949
