# Supplementary material for: Lower promoter activity of the ST8SIA2 gene has been favored in evolving human collective brains
Source: PLoS One. 2021 Dec 16;16(12):e0259897. doi: 10.1371/journal.pone.0259897 (PMC8675693; doi:10.1371/journal.pone.0259897)
Supplement: S5 Fig — The phylogenetic tree was constructed by the Neighbor-Joining method with the number of differences. Bootstrap values of more than 50% from 1,000 replications are shown on the tree branches. (PDF) [file pone.0259897.s005.pdf]

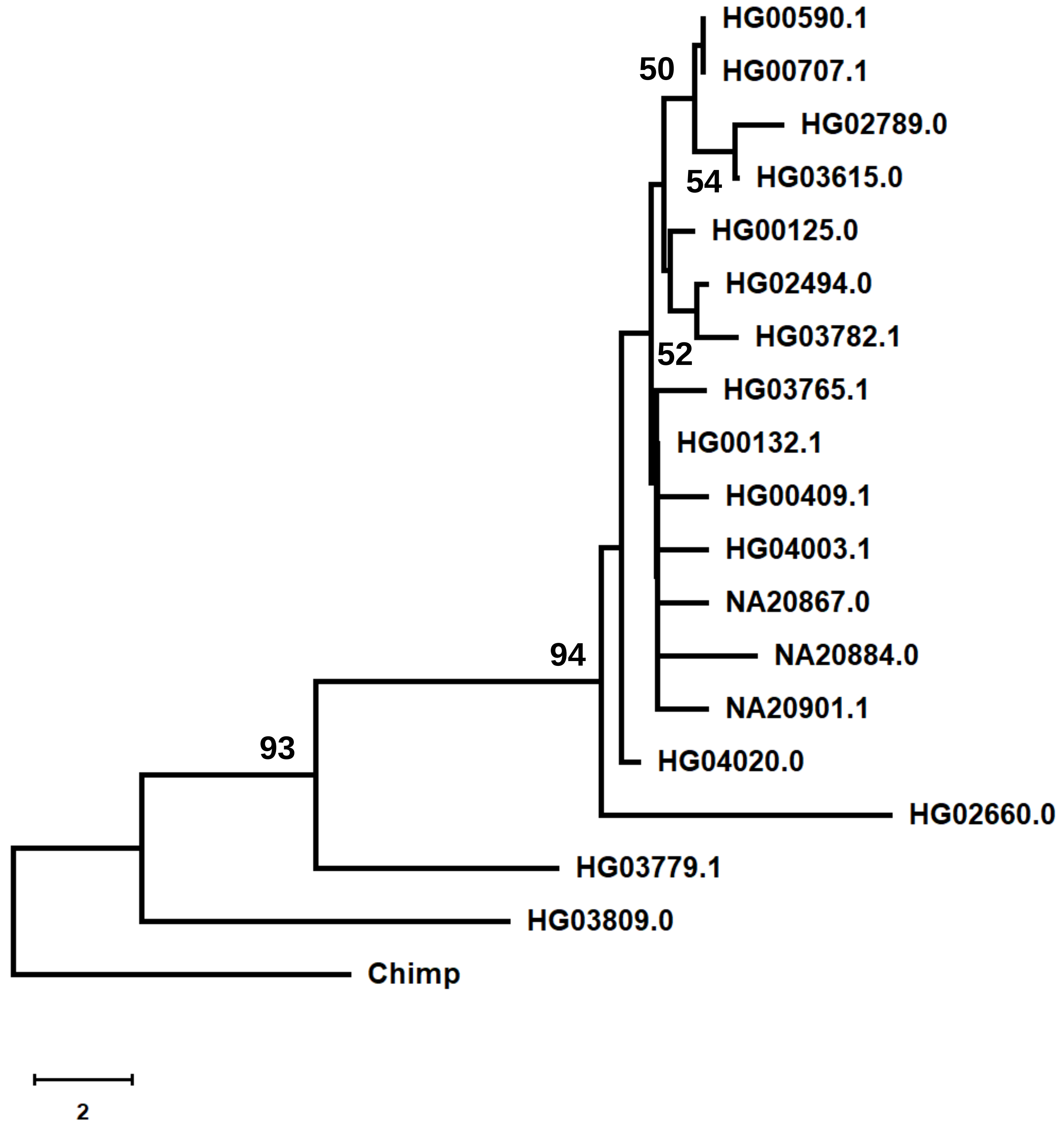
HG00590.1
HG00707.1
HG02789.0
HG03615.0
HG00125.0
HG02494.0
HG03782.1
HG03765.1
HG00132.1
HG00409.1
HG04003.1
NA20867.0
NA20884.0
NA20901.1
HG04020.0
HG02660.0
HG03779.1
HG03809.0
Chimp
50
54
52
94
93
2
