# Supplementary material for: Lower promoter activity of the ST8SIA2 gene has been favored in evolving human collective brains
Source: PLoS One. 2021 Dec 16;16(12):e0259897. doi: 10.1371/journal.pone.0259897 (PMC8675693; doi:10.1371/journal.pone.0259897)
Supplement: S3 Table — (PDF) [file pone.0259897.s016.pdf]

S3 Table. Distribution of the TCT haplotypes*

	AFR							EUR					SAS					EAS					AMR				
Haplotype	ACB	ASW	ESN	GWD	LWK	MSL	YRI	CEU	FIN	GBR	IBS	TSI	BEB	GIH	ITU	PJL	STU	CHB	CHS	CDX	JPT	KHV	CLM	MXL	PEL	PUR	Total
HG00097.1	13	15	7	9	13	6	7	76	75	72	94	100	30	43	36	49	30	66	75	45	48	45	52	36	34	70	1146
HG00105.1	1	1		1	3			34	21	27	36	33	23	33	27	16	43	8	10	15	10	24	31	7	6	22	432
HG00130.0	4	2						11	5	8	6	6	21	16	10	12	17	10	16	15	3	10	1	3		3	179
HG00096.0	4	1	1	2	2	1	2	3	2	5	12	10	7	9	7	11	8	14	7	6	9	10	2	13	9	9	166
HG01556.0	14	5	20	23	16	14	15																1		3		111
HG00131.0	2	2		1	1			10	6	6	7	9	1	5	3	6	1						11	1		8	80
HG00129.1	2	1						6	14	2	4	1	4	5	5	9	10	3				1	3	2	1	3	76
HG01254.1	12	11	20	9	4	3	13																1				73
HG01073.0	4	3	13	20	4	6	13																1		1	2	67
HG00109.1								8	28	7	7	6		1			1						2	3		1	64
HG01149.0	10	2	15	6	5	5	15																3				61
HG01589.0													12	11	12	14	12										61
HG00732.0	3	3	10	14	3	12	7																4	1	1	1	59
HG00149.0	1	1						6	9	3	3	3	7	4	5		8					1	2	2		1	56
HG00100.0	1	1						7	1	10	4	1	2	5	3	8	6						2			4	55
HG01047.1	12	2	3	9	3	5	11																			4	49
HG01188.0																		3			1		5	8	23	1	41
HG02127.1													5	8	15	4	6					2					40
HG00117.1								1	2	4	3	7		1	5	1	1	2		2			2	1		3	35
HG00114.0										4	1	7	5	1	3	4	3	1							2		31
HG00100.1					1			3	1	3	2	4			1	1	1	3	2	2	1		1		1	1	28
HG00118.0						1		2	2	2	1												1	2		14	25
HG00096.1	1	1						2	1	2	3	1	1			1	1			1	3			3	2	1	24
HG03634.1								1				1	1	3	6	2	4							2			20
HG01912.0	3		9		2	1	4																				19
HG00099.1								2	2	4	2	2				4										1	17
HG00110.1	2	1						2		2	4	2		1				1	1		1						17
HG02485.1	2	2	2	5	2	1	1																				15
HG00140.1								3	3	1	2	2			1											2	14
HG00099.0										2			2	4	1	3											12
HG00123.1										2		1		1	1	1	4				1					1	12
HG00102.0								1		2	3									1		1		2		1	11
HG00536.1																		6	2	1	1						10
HG01311.1	1		1		2	1	3																			1	9
HG00339.0								1	2			1											1	1		2	8
HG02642.1				3		4	1																				8
HG03686.1														2	4		2										8
HG01354.1								3			1	2											1				7
HG02058.1					1			1										1			1	3					7
HG03836.0													1	1	4		1										7
HG01991.1								1				1		1			1	1							1		6
HG02687.0															1	1	4										6
HG00182.0									3							1	1										5
HG02051.1	4			1																							5
HG02442.1	2				1		2																				5
HG00334.1	1								2														1				4
HG01284.0	1		1		1																		1				4
HG01389.1											2												1		1		4
HG02734.1													1	2		1											4
HG00106.0		1								1		1															3
HG00171.1									1	1													1				3
HG00259.0									1	1	1																3
HG00266.1									1																	2	3
HG00640.0					2																					1	3
HG01121.0																	1				1		1				3
HG01357.1											1				1								1				3
HG01438.0					2																		1				3
HG01485.0								1													1		1				3
HG01871.1																						3					3
HG02130.1																		1				2					3
HG02600.0																1	2										3
HG02793.1												1	1			1											3
HG03129.0			2				1																				3
HG03301.0			1		2																						3
NA18945.1																					3						3
HG00133.1		1								1																	2
HG00145.0									1	1																	2
HG00306.1								1	1																		2
HG00553.1																										2	2
HG01518.0											1														1		2
HG01631.1											2																2
HG02339.0	1			1																							2
HG02574.0		1		1																							2
HG02654.0													1			1											2
HG02896.0				1			1																				2
HG02974.0			2																								2
HG03015.1																1		1									2
HG03052.1						2																					2
HG03428.1						1										1											2
HG03458.1						2																					2
HG03717.1															2												2
HG03730.0															1		1										2
HG03742.1													1		1												2
NA18520.0							2																				2
NA20503.1												1		1													2
HG00154.1										1																	1
HG00171.0									1																		1
HG00178.1									1																		1
HG00238.0										1																	1
HG00251.1										1																	1
HG00284.1									1																		1
HG00327.1									1																		1
HG00457.0																			1								1
HG00743.1																										1	1
HG01077.1																										1	1
HG01108.0																										1	1
HG01190.0																										1	1
HG01251.1																							1				1
HG01413.0																										1	1
HG01519.1											1																1
HG01628.0											1																1
HG01631.0											1																1
HG01866.1																						1					1
HG01977.0																									1		1
HG02178.1																				1							1
HG02232.1											1																1
HG02236.0											1																1
HG02277.1																									1		1
HG02427.0	1																										1
HG02502.0	1																										1
HG02635.1				1																							1
HG02757.0				1																							1
HG02811.0				1																							1
HG02944.1			1																								1
HG03006.1													1														1
HG03018.1																1											1
HG03160.0			1																								1
HG03209.1						1																					1
HG03224.1						1																					1
HG03228.1																1											1
HG03229.1																1											1
HG03436.1						1																					1
HG03556.1						1																					1
HG03578.0						1																					1
HG03681.0																	1										1
HG03697.1																	1										1
HG03706.0																1											1
HG03713.0															1												1
HG03722.1															1												1
HG03729.1															1												1
HG03752.1																	1										1
HG03765.0																1											1
HG03920.0													1														1
HG03965.0															1												1
HG04001.0															1												1
HG04134.0													1														1
HG04155.1													1														1
HG04194.1													1														1
NA07048.0								1																			1
NA07051.0								1																			1
NA11832.0								1																			1
NA11992.1								1																			1
NA18549.1																		1									1
NA18605.1																		1									1
NA18631.1																		1									1
NA18757.0																		1									1
NA19024.0					1																						1
NA19146.0							1																				1
NA19700.1		1																									1
NA19771.1																								1			1
NA19904.1		1																									1
NA19920.1		1																									1
NA19982.1		1																									1
NA20274.1		1																									1
NA20318.0		1																									1
NA20334.0		1																									1
NA20503.0												1															1
NA20542.0												1															1
NA20783.1												1															1
NA20786.0												1															1
NA20798.1												1															1
NA20887.0														1													1
NA21129.1														1													1
Total	103	64	109	109	71	70	99	190	188	176	207	208	131	160	160	159	172	125	114	89	84	103	136	88	88	166	3369

* SNPs with minor allele frequency of 1% or over in total AMH population were used.
